# A Late Meal Timing Pattern Is Associated with Insulin Resistance in European Children and Adolescents

**DOI:** 10.1155/2024/6623357

**Published:** 2024-03-01

**Authors:** Timm Intemann, Leonie H. Bogl, Monica Hunsberger, Fabio Lauria, Stefaan De Henauw, Dénes Molnár, Luis A. Moreno, Michael Tornaritis, Toomas Veidebaum, Wolfgang Ahrens, Antje Hebestreit

**Affiliations:** ^1^Leibniz-Institute for Prevention Research and Epidemiology—BIPS, Bremen 28359, Germany; ^2^Department Nutrition and Dietetics, Faculty of Health Professions, Bern University of Applied Sciences, Bern 3012, Switzerland; ^3^Institute for Molecular Medicine Finland (FIMM), University of Helsinki, Helsinki, Finland; ^4^School of Public Health and Community Medicine, Institute of Medicine, Sahlgrenska Academy, University of Gothenburg, Gothenburg 40530, Sweden; ^5^Institute of Food Sciences, National Research Council, Avellino 83100, Italy; ^6^Department of Public Health and Primary Care, Ghent University, Ghent 9000, Belgium; ^7^Department of Pediatrics, Medical School, University of Pécs, Pécs 7623, Hungary; ^8^GENUD (Growth Exercise, Nutrition and Development) Research Group, Instituto Agroalimentario de Aragón (IA2), Instituto de Investigación Sanitaria Aragón (IIS Aragón), Centro de Investigación Biomédica en Red Fisiopatología de la Obesidad y Nutrición (CIBERObn), University of Zaragoza, Zaragoza 50009, Spain; ^9^Research and Education Institute of Child Health, Strovolos 2035, Cyprus; ^10^Department of Chronic Diseases, National Institute for Health Development, Tallinn 11619, Estonia

## Abstract

Meal timing has been associated with metabolic markers in adults, but not in children or adolescents. The aim of this study was to investigate associations of meal timing patterns (MTPs) with insulin resistance (IR) and triglyceride levels in children and adolescents. In this cross-sectional study, we included 2,195 participants aged 8–15 years from the European I.Family study (2013/14). Habitual diet exposures were derived using 24-hr dietary recalls and HOMA-IR, HbA1c, and triglycerides were used as metabolic outcome variables. We applied *k*-means cluster analysis on five dietary exposures (energy proportion in the morning and evening, eating window, pre-sleep fasting and eating frequency), which revealed the following three MTPs: “early-often”, “late-long” and “late-infrequent-short”. We used linear mixed models to estimate the associations between MTPs and the *z*-scores of the metabolic outcome variables. The association analysis revealed differences between MTPs in HOMA-IR but not in HbA1c or triglyceride *z*-scores. The “late-infrequent-short” pattern was associated with a 0.19 (95%-CI: (0.01, 0.36)) higher HOMA-IR *z*-score compared to the “early-often” pattern in the model adjusted for age, BMI *z*-score, education, sex, country, and family membership. These findings suggest that the timing of meals may influence IR already in childhood and adolescence. Therefore, the time of meals should be considered in future nutrition research and dietary advice for children and adolescents.

## 1. Introduction

Chrono-nutrition is an emerging field of research that focuses on the interplay between nutrition, circadian rhythm (CR), and metabolism [1]. CR is an internal process that regulates the sleep–wake cycle and repeats roughly every 24 hr. It controls a wide variety of physiological events, including metabolism, immune response, inflammation, and hormone secretion. For example, the blood concentration of cortisol, the most important glucocorticoid is low during the night, peaks in the morning, and decreases again throughout the day [2]. Many metabolic pathways and functions, including lipid metabolism, blood pressure, and glucose homoeostasis vary according to the time of the day. For example, among healthy individuals, endogenous glucose production was lower and insulin sensitivity is higher at breakfast (7 am) than at lunch (1 pm) or at dinner (7 pm) [3]. Further, diet-induced thermogenesis is lower in the evening than in the morning [4]. CR is also affected by external cues, such as light, the timing of meals and fasting [5], therefore providing a plausible biological process linking meal timing to metabolic health.

Current trends in meal timing among both children and adults suggest that a greater proportion of energy intake is shifted from earlier to later in the day [6, 7]. Several animal and human studies suggest that this trend could be potentially detrimental for health. Studies among rodents have shown that feeding during the normal sleep phase leads to changes in the hypothalamic areas, which promote weight gain and metabolic alterations as compared to those fed during the normal activity phase, despite similar or even a lower food intake [8, 9]. In humans, the evening chronotype has been associated with adverse cardiovascular health compared to the intermediate or morning types [10]. For example, in adult night shift workers, studies have reported an increased risk of a range of metabolic disorders and diseases, possibly reflecting the fact that they skip more meals and consume more food at night compared to day workers, which together with sleep deprivation and circadian desynchronization could contribute to their increased risk [11].

In children and adolescents, few studies on meal timing patterns (MTPs) and metabolic health are available and have mainly considered skipping breakfast. A systematic review of the associations between skipping breakfast and cardiometabolic risk factors found consistent evidence to support the hypothesis that a shift of energy intake toward later in the day is associated with an atherogenic lipid profile, insulin-resistance (IR), and the metabolic syndrome [12]. In a 2017 scientific statement, the American Heart Association (AHA) recommended that the impact of meal timing, particularly related to the evening meal, deserves further studies. In addition, in their statement the AHA emphasized that there has been limited published research investigating meal patterns and meal frequency among children and adolescents and further, the related body weight implications and other metabolic risk factors [13].

Thus, we aimed to investigate the cross-sectional association of MTPs with IR and triglycerides in European children from eight European countries. Since many variables related to meal timing are closely interrelated, we applied cluster analysis to identify subgroups of children with similar MTPs with regard to the timing of energy intake throughout the day, the eating window within a day, hours of pre-sleep fasting and the eating frequency of all meals and snacks.

## 2. Materials and Methods

### 2.1. Study Participants

The data for this investigation come from the population-based I.Family study [14], including healthy participants. The overriding aim of the I.Family study was to identify determinants of food choice, lifestyle, and health in European children and adolescents and their families. The examinations were conducted in 2013/2014 in families from eight European countries (Belgium, Cyprus, Estonia, Germany, Hungary, Italy, Spain, and Sweden). For this analysis we used data of 2,195 children and adolescents from 8 to 15 years with laboratory and covariate information and with a dietary report of at least 500 kcal energy intake.

The study was approved by the ethic committees responsible in each country and has been conducted according to the guidelines laid down in the 1964; Declaration of Helsinki and its later amendments. Parents provided written informed consent, while children aged 12 and over gave simplified written consent. Younger children gave oral consent for examinations and sample collection. The children and adolescents and their parents were offered the possibility to consent to single components of the study while abstaining from the others.

### 2.2. Questionnaires, Anthropometric Measurements, and Blood Parameter

Parents reported the age and sex of their children as well as their highest educational level according to the International Standard Classification of Education (ISCED) [15]. As part of a dietary questionnaire [16], parents, children, and adolescents aged 12 and over reported the frequency for breakfast, lunch, dinner, and snacks (five answer options from “Never” to “Daily”). Based on this information, the total meal frequency was calculated. Parents were also asked to report the daily nocturnal sleep duration and napping times at weekends (WE) and weekdays (WD). Based on this information the total daily sleep duration was calculated as follows:

(1) $$\begin{array}{c} {\text{Sleep}}_{\text{total}}=2/7\left({\text{Sleep}}_{\text{nocturnal WE}}+{\text{Sleep}}_{\text{nap WE}}\right)+5/7\left({\text{Sleep}}_{\text{nocturnal WD}}+{\text{Sleep}}_{\text{nap WD}}\right). \end{array}$$

Body height and weight were taken by trained personnel following standard operation procedures [17, 18]. Height was measured using a Seca 225 stadiometer (Seca GmbH and KG, Birmingham, UK), while body weight was assessed in fasting state in light clothing (using an adapted TANITA BC 420 SMA for children ≤6 years, TANITA BC 418 MA for participants >6 years, TANITA Europe GmbH, Sindelfingen, Germany). Body mass index (BMI) was calculated by dividing body weight in kilograms by the squared body height in meters. Age- and sex-specific BMI *z*-scores were derived and categorized in weight status groups (underweight, normal weight, overweight, and obese) consistently to children and adolescents from all included countries according to the extended IOTF criteria [19]. Pubertal status was assessed based on the onset of voice mutation in boys and menarche in girls which was asked during examination. Parents were asked to report children's chronic diseases. Thus, also information on type 2 diabetes is available. In a subsample, accelerometery data were used to assess the physical activity (PA) of participants. Moderate-to-vigorous PA (MVPA) was derived as described elsewhere [20].

Fasting blood samples were collected and assessed for glucose, insulin, glycated haemoglobin (HbA1c), and triglycerides following standard operation procedures [21]. Glucose and insulin values were used to calculate the homoeostasis model assessment for IR (HOMA-IR). As levels of these blood parameters change during childhood, sex- and age-specific *z*-scores were derived based on the I.Family study data as described previously for the IDEFICS study [22, 23].

### 2.3. Dietary Exposure Assessment

Dietary exposures were assessed using an online 24-hr dietary recall (24HDR) assessment program, called “Self-Administered Children, Adolescents and Adult Nutrition Assessment” (SACANA) [24], based on the validated SACINA offline version [25]. The SACANA instrument has been validated [26] and the repeatability as well as the plausibility of the instrument has been assessed [27]. Children and adolescents were asked to recall their diet and to enter the type and the amount (g) of all foods and beverages consumed during the previous day, starting with the first intake in the morning. Standardized photos were used to assist with accurate estimation of portion size. Furthermore, the time point of meals, waking up times and bedtimes were requested. Parents were asked to proxy report for younger children and/or assist children in filling in the 24HDR, especially those below the age of 11 years. Participants were invited to complete at least three 24HDR, the first of which was to be completed on the day of blood collection in order to measure the dietary nutrients consumed on the day before the blood collection (45% reported the first 24HDR on the day of blood collection, 74% during the following 10 days). However, the availability of repeated 24HDRs varied in this study sample (49.4% provided one recall, 27.3% two recalls, and 23.3% three or more recalls).

The individual daily exposures were calculated based on the 24HDR and country-specific food composition tables [28]. These included the total daily energy intake (EI), the EI proportion between 5 and 11 am (in %), the EI proportion between 5 pm and 12 am (in %), and the EI of healthy food according to Hebestreit et al. [29], the time between first and last meal (eating window), the time between last meal and bedtime (pre-sleep fasting, in hours/day) and the total number of consumed meals and snacks during 24 hr (eating frequency). Usual energy proportion between 5 and 11 am (%) was calculated as ratio of usual EI between 5 and 11 am (kcal) and usual total EI (kcal) times 100 (analogously for usual energy proportion between 5 pm and 12 am). Only reports with an energy intake of less than 500 kcal were excluded from the analysis as recommend by Rhee at al. [30]. The time windows 5−11 am and 5 pm-midnight were defined based on an assessment of the country-specific meal times for breakfast, lunch, and dinner (Table S1).

Since measurement error is inevitable, correction methods are strongly advised to reduce bias in effect estimates when analyzing 24HDR data [31]. Therefore, usual dietary exposures were estimated based on the National Cancer Institute (NCI) method [32, 33]. The underlying error model considered the daily variation in diet, covariates, and the skewness of the intake distributions. Usual dietary exposures were estimated for participants stratified by sex and considering the day of the week (weekend vs. weekday), age, ISCED, and BMI *z*-score following the regression calibration approach [33] and additionally considering the HOMA-IR *z*-score following the multiple imputation approach [34]. Furthermore, the daily EI was used to classify each recall day as misreported or plausibly reported according to the Goldberg cutoffs [35] adapted for children [36] and adolescents [29].

### 2.4. Statistical Analysis

The descriptive analysis was performed for the complete study population and stratified by MTPs. We calculated means and standard deviations (SDs) of the outcomes, the continuous covariates and exposures. For the categorical variables sex, country, weight status, ISCED, and misreporter status the numbers and percentages were determined. Furthermore, we calculated the means and SDs of HOMA-IR, HbA1c, and triglyceride *z*-scores by sex, country, ISCED, and weight status.

To analyze the association between MTPs and the outcomes HOMA-IR, HbA1c, and triglycerides *z*-score a so-called three stage approach was applied, which is based on the multiple imputation [34]. The method reduces the bias due to categorization based on cluster analysis using multiple continuous error-prone exposures. In brief, the approach is based on three stages as the name implies:

1. Calculating the usual exposures (energy % between 5 and 11 am, energy % between 5 pm and 12 am, eating window, pre-sleep fasting and eating frequency) as described in Section 2.3;
2. Deriving the MTPs using k-means cluster analysis based on the *z*-score of the usual exposures from stage [1]; and
3. Fitting a regression model using the derived MTP as exposures.

To account for misclassification of the MTPs, we generated individual random exposures given individual covariates, individual reports, and the fitted-error model from Stage 1 considering daily variation. Then we classified everyone to a MTP based on the generated individual exposures and the classification function from Stage 2. In Stage 3, the regression model is fitted to estimate the association between the outcome and MTP. We repeated this procedure 500 times to account for variability of the individual exposures due to daily variation. The resulting effects parameters were averaged to calculate the final MI estimate. For statistical details of this approach refer the study by Intemann and Pigeot [34]. Details of the cluster analysis can be found in the supplementary materials. In Stage 3, linear mixed models were used to investigate the association between the MTPs and the outcomes HOMA-IR, HbA1c or triglyceride *z*-scores, respectively. The basic model was adjusted for age, BMI *z*-score, ISCED, sex, and country as well as family membership as random effect to account for sibling structure in the data. In sensitivity analyses, three further models considering (1) puberty status, (2) usual EI of healthy foods, and (3) a fully adjusted model including usual EI, usual healthy food intake (HFI), and sleep duration were investigated. The 95% confidence intervals (CIs) were calculated using Rubin's rule.

To analyze the association between the single mealtime exposures and the outcomes HOMA-IR, HbA1c, and triglyceride *z*-score, a best subset selection was conducted to select only the most important variables and to avoid multicollinearity between exposures. For this purpose, we selected exposures from the five usual exposures derived following the regression calibration approach (as described in Section 2.3), while the same adjustment variables as before were used, i.e., these covariates were fixed. Then, the resulting exposures were used in linear mixed models to estimate the exposure coefficients. The CIs were estimated with the Bootstrap method using 1,000 bootstrap samples drawn with replacement.

The analysis of MTPs and associations with health outcomes was repeated for the subsample of participants with plausible dietary reports for the purpose of sensitivity analysis (*N* = 1,755). Furthermore, in a sensitivity analysis we investigated a model using MVPA and a model using an interaction term of MTP and overweight/obesity (yes, no) as additional covariate. We used the statistical software R 3.6.2 [37] for the statistical analysis, especially the packages lme4 [38], mclust [39], and leaps [40], except the usual intake estimation for which SAS was used (version 9.3; SAS Institute, Cary, NC, USA).

## 3. Results

### 3.1. Descriptive Analysis

The results of the descriptive analyses are presented in Tables 1 and 2. The sex ratio was almost balanced. Most children and adolescents in the study population were from Italy (27%) and Estonia (25%) and the fewest from Hungary (4%) and Spain (5%). More children and adolescents were of normal weight (66%) than overweight and obese (27%). High-parental ISCED was slightly more frequent than low-parental ISCED. According to the Goldberg cutoffs, 20% of the study population were classified as misreporters. On average the children and adolescents reported an intake of approximately 1,600 kcal, whereas more unhealthy rather than healthy food was consumed. The energy proportion was lower in the morning (26%) than in the evening (36%). On average, children and adolescents ate 4.2 meals in 11.5 hr of eating window and went to bed 2.5 hr after the last meal.

Table 2 gives the mean HOMA-IR, HbA1c, and triglyceride *z*-scores by sex, country, ISCED, and weight status groups. Boys and girls had the same mean *z*-score values for all three measures. The children and adolescents showed different *z*-scores on all measures by country. Mean HOMA-IR *z*-scores ranged from −0.4 in Swedish to 0.5 in Italian participants, mean HbA1c *z*-scores ranged from −0.5 in Swedish to 0.3 in Belgian participants, and mean triglycerides *z*-scores ranged from −0.3 in Spanish to 0.3 in Hungarian participants. In addition, HOMA-IR *z*-score was higher with a higher weight status and higher in the low-ISCED group than in the high-ISCED group.

### 3.2. Description of Meal Timing Patterns

**Figure 1:**
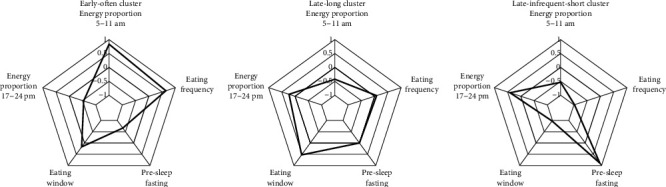
Meal timing patterns of children and adolescents: “early-often,” “late-long,” and “late-infrequent-short”. Cluster means of the three meal timing patterns are shown (for details see Table 3). The scale ranges from −1 to 1 with additional tick lines for −0.5, 0, and 0.5.

The three resulting MTPs from the k-means cluster analysis can be found in Figure 1 and Table 3. We named the three patterns as follows: “early-often” (*N* = 791), “late-long” (*N* = 819), and “late-infrequent-short” (*N* = 585). The “early-often” pattern is characterized by the highest energy proportion between 5 and 11 am, the lowest energy proportion between 5 pm and 12 am, a below-average eating window, the lowest pre-sleep fasting time, and the highest mean eating frequency. In contrast, the “late-long” pattern is characterized by a lower than average energy proportion between 5 and 11 am, a higher than average energy proportion between 5 pm and 12 am, the longest eating window, an average pre-sleep fasting time and an average eating frequency. The “late-infrequent-short” pattern is characterized by the lowest energy proportion between 5 and 11 am, the highest energy proportion between 5 pm and 12 am, the shortest eating window, the longest pre-sleep fasting phase and the lowest eating frequency. In sensitivity analyses based on only plausible reports, we identified very similar MTPs (Figure S1).

The participants of the “late-infrequent-short” pattern had the highest HOMA-IR, HbA1c, triglycerides, and BMI *z*-scores and they were older than the children and adolescents of the other MTPs (Table 1). They reported the lowest EI overall and this was true for healthy as well as unhealthy foods. According to the Goldberg cutoffs, participants of this MTP misreported the most frequently (36%). In contrast, participants of the “early-often” pattern had the lowest HOMA-IR, triglycerides, and BMI *z*-score. They reported the highest EI and the highest intake of healthy food. Participants of this MTP also misreported the least frequently (9%). The participants of the “late-long” pattern had HOMA-IR, triglycerides, and BMI *z*-score values close to the study population's mean values and between the extreme values of the other two MTPs. This group had the lowest HbA1c *z*-scores. They reported the highest intake of unhealthy food and had a proportion of misreporters close to the whole study population (19%).

### 3.3. Associations between Meal Timing Patterns and Health Outcomes

The basic linear mixed model for HOMA-IR *z*-score revealed that children and adolescents of the “late-infrequent-short” pattern had a 0.19 (95% CI: (0.01, 0.36)) and of the “late-long” pattern a 0.06 (95% CI: −0.09, 0.21)) higher HOMA-IR *z*-score as children and adolescents of the “early-often” pattern (Table 4). The basic linear mixed models for HbA1c and triglycerides *z*-scores revealed only very small differences between MTPs. Regarding HbA1c *z*-scores, children and adolescents of the “late-long” and the “late-infrequent-short” patterns both had only a difference of −0.02 (95% CIs: (−0.15, 0.11) and (−0.17, 0.14), respectively) to children and adolescents of the “early-often” pattern. Children and adolescents of the “late-long” pattern had a 0.02 (95% CI: (−0.11, 0.15)) and of the “late-infrequent-short” pattern had a 0.05 (95% CI: (−0.11, 0.21)) higher triglycerides *z*-score as compared to children and adolescents of the “early-often” pattern. The sensitivity analysis with additional adjustment for puberty status, for EI and intake of healthy foods and further adjustment for sleep duration revealed only negligible changes in effect estimates and CIs. When the sample was restricted to only the subsample with plausible energy intake reports (according to Goldberg), the results were very similar to the main analysis (Table S2).

### 3.4. Associations between Selected Single Meal Timing Exposures and HOMA-IR z-Score

The best subset analysis selected models considering the three exposures: eating window, energy proportion 5−11 am, and pre-sleep fasting as best-fitting models (Table 5). The corresponding estimated *β* coefficients were −0.16 (95% CI: (−0.35, −0.11)), −0.01 (95% CI: (−0.02, 0.00)), and −0.15 (95%-CI: (−0.37, 0.00)), respectively, which highlights the importance of the eating window. The sensitivity analysis considering further covariates (EI, intake of healthy food and additionally sleep duration) showed only a slightly higher *β* estimate for the variable eating window. In the corresponding analysis for the outcomes HbA1c and triglycerides *z*-score only one exposure (energy proportion 5−11 am or energy proportion 5 pm−12 am) was selected for the best-fitting model and the corresponding estimated coefficients were between 0 and 0.01 (Tables S3 and S4). The analysis of the subsample with plausible reports did not reveal large differences to the main analysis (Tables S5–S7).

## 4. Discussion

The present study reported the association between meal timing patterns with triglycerides and markers of IR in children, showing higher mean values of HOMA-IR in children who belonged to the “late-infrequent-short” pattern as compared to those belonging to the “early-often” pattern. The “late-infrequent-short” pattern was characterized by a lower eating frequency, a shorter eating window, longer pre-sleep fasting time, and a higher energy consumption in the evening, as compared to the “early-often” pattern. We also reported descriptive country differences in MTPs, showing that children from Central European countries and Sweden belonged most frequently to the “early-often” pattern, while children from Cyprus, Italy, and Spain were most often assigned to the “late-long” pattern. Children from Estonia, in contrast, were mostly categorized by the “late-infrequent-short” pattern.

To our knowledge, there are no published reports on MTPs in European children. Some studies in adults have reported marked differences in eating frequency, overnight fasting, and time between single eating occasions between European countries [41, 42]. The European Prospective Investigation into Cancer and Nutrition (EPIC) study investigated the timing of eating in 10 European countries and reported a south–north gradient for the timing of eating, with later consumption of meals and snacks in Mediterranean countries compared with Central and Northern European countries. This is in line with our observation in European children, apart from Estonia, which most frequently belonged to the MTP “late-infrequent-short” [41].

To date, only few studies have investigated the associations between MTPs and metabolic variables in children and adolescents. The most explored meal timing variable in the literature is skipping breakfast, and systematic reviews report an increased risk of becoming overweight or obese compared with not skipping breakfast in this age group [12, 43, 44]. Skipping breakfast has also been associated with an increased risk of IR and metabolic syndrome [45–47]. However, there are data to suggest that observational studies focusing exclusively on skipping breakfast are subject to confounding by other meal pattern variables, in that individuals who skip breakfast have a lower eating frequency [48], more energy intake at lunch and during afternoon and evening snacks [49], and a lower diet quality [50]. In addition, skipping breakfast has been associated with going to sleep late and with short sleep duration in young adults [51]. Since many of these variables are closely interrelated and tend to co-occur in the same individuals, we applied cluster analyses, a data-driven approach to create MTPs of individuals sharing similar habits. We found that children belonging to the MTP characterized by low EI in the morning, high EI in the evening, short eating window during the day, and a low eating frequency had higher IR, measured by HOMA-IR, compared to children belonging to the MTP characterized by high EI in the morning, low EI in the evening, a longer-than-average eating window, and a higher eating frequency.

Previous studies in children and adolescents have mainly examined meal patterns in relation to overweight and obesity. In children and adolescents aged 4–18 years from the UK, the timing of the evening meal was not related to the risk of overweight or obesity and neither with energy intake [52]. Some studies have reported an association between meal skipping and overweight and obesity in children [53] or an inverse association between eating frequency and adiposity [54], while other studies have reported a positive association between both snacking and eating frequency with a higher risk of overweight and abdominal obesity in children [55]. A population sample of Finnish girls and boys aged 6–8 years showed that skipping main meals is associated with an increased risk of having overweight or obesity, increased body fat percentage, and higher waist and hip circumferences in children, while no association was seen with the number of snacks [56]. The macronutrient composition of meals may also deserve further investigation, as a previous study using the same cohort has shown that short sleep duration and high carbohydrate-starch intake in the morning are positively associated with BMI *z*-scores in children aged 2–9 years [57].

Previous experimental studies among adults have explored the effects of meal timing on glucose control and insulin secretion and those studies point toward the fact that having the largest energy load in the evening may contribute to the metabolic syndrome through deterioration in postprandial glucose and insulin, independent of the glycaemic index [58, 59]. Our findings suggest that eating earlier in the day and, having a longer eating window, and a higher eating frequency is related to increased insulin sensitivity in children. The longer eating opportunity is likely a function of being an early riser with an earlier opportunity to eat. The underlying mechanisms may be related to the liver CR, since breaking the overnight fast by having breakfast has been shown to reset the biological liver clock in mice [60]. Furthermore, breakfast skipping adversely affects clock and clock-controlled gene expression in both healthy individuals and people with Type 2 diabetes [61].

Much recent research attention has focused on time-restricted eating in adults that involves limiting food intake to an 8-hr window during the day. As fasting stimulates autophagy, it is hypothesized that prolonged fasting periods may be effective in improving the insulin sensitivity in prediabetic adults [62]. Our study does not align with this hypothesis, as we found that a longer eating window during the day was related to increased insulin sensitivity in children and adolescents. This becomes clear when comparing the HOMA-IR of the “early-often” pattern and “late-infrequent-short” pattern as well as when looking at the results of the best subset selection analysis, which emphasizes the importance of the eating window. However, our findings do not allow a direct comparison with studies of intermittent fasting, because even in the MTP with the shorter eating window, the mean eating window was 11 hr.

Several strengths and limitations of our study merit consideration. The main limitation is the use of cross-sectional data, which do not allow for the investigation of causal relationships between exposure and outcome. While objectively measured weight, height, and markers of IR are a strength, we relied on self-reported data for diet and meal patterns, with many well-known limitations [63]. However, we conducted a sensitivity analysis excluding misreporters which showed similar MTP and effect estimates. Further, in the main analysis we followed the recommendations for analyzing self-reported dietary intake data [64, 65] and did not exclude misreporters but adjusted for BMI instead. Moreover, we have adjusted for self-reported pubertal status, but as the children following the “late-infrequent-short” pattern were older and more likely in puberty, the observed worse metabolic status in this group may still be influenced by pubertal insulin resistance due to residual confounding. The strengths of our study include the use of cluster analyses considering five interrelated dietary exposures, which closely reflect the real-life setting, and similarly the use of dietary pattern analyses for dietary intake [66]. The misclassification in the MTP due to daily variation in daily report and the resulting bias in the effect estimates were reduced using the newly developed three stage approach based on the multiple imputation correction method [34]. The approach even allows for calculating CIs considering the extra variability in the estimated parameters of the underlying measurement error model. In the additional best subset selection approach, we also followed a rigorous measurement error correction approach. Further, in using this approach for the calculation of the CIs, the extra variability was considered by using the bootstrap as recommended by Keogh et al. [67].

Finally, the I.Family study allows a deep investigation of MTPs and the association with insulin resistance in children and adolescents across Europe. The large sample includes data collected and processed in a standardized manner in eight European countries.

Our finding that children who eat late, infrequently, and during a short period of time during the day tend to have higher HOMA-IR values has an important public health implication. Future nutrition educational programs should increasingly focus on the timing of meals, in addition to the quality of meals. Further, children and adolescents in all countries should have access to affordable and nutritious breakfast and lunches at kindergarten and schools, in order not to postpone mealtimes.

## 5. Conclusion

To conclude, scientific evidence in children on meal timing and meal patterns in relation to metabolic health is sparse, with the exception of several reports that have examined skipping breakfast. Our research provides novel evidence that shifting energy intake to earlier in the day, within a 4–5 eating occasions per day pattern, is related to increased insulin sensitivity in children. This is in line with the commonly recommended three main meals and 1–2 snacks per day pattern. This work indicates that considerably more work will need to be done in the future to determine the longitudinal relationship between MTPs and IR in children and to study the joint effects of meal timing and the timing of other behaviors, such as sleep and physical activity.

## Figures and Tables

**Table 1:** Characteristics of the complete study population and of the obtained meal timing patterns (mean and standard deviation (SD); number (*N*) and percentages (%)).

	Complete study population		Meal timing patterns						
			“Early-often”		“Late-long”		“Late-infrequent-short”		
	Mean	SD	Mean	SD	Mean	SD	Mean	SD	
HOMA-IR *z*-score	0.19	1.18	−0.09	1.2	0.17	1.1	0.59	1.1	
HbA1c *z*-score	−0.04	0.97	−0.01	1	−0.09	1	−0.01	0.9	
Triglycerides *z*-score	0.01	1.01	−0.13	1	0.01	1	0.18	1	
Age (yrs)	11.75	1.78	10.5	1.4	12.27	1.6	12.7	1.5	
BMI *z*-score	0.59	1.11	0.25	1.1	0.66	1.1	0.95	1.1	
Energy (kcal)	1,602.7	243	1,641	226	1,637.7	240	1,502	241	
Unhealthy food (kcal)	1,122	229	1,126	220	1161.9	235	1,061	221	
Healthy food (kcal)	483.2	123	516.7	121	476	115	447.9	124	
Bed time (hh:mm)	22 : 18	1.1	21 : 42	0.9	22 : 30	1	22 : 48	1	
Get up time (hh:mm)	7 : 42	1.1	7 : 30	0.9	7 : 36	1.1	8 : 00	1.2	
Total sleep duration (hr)	9.3	1	9.6	0.8	9.1	1.1	9.1	1.1	
Moderate-to-vigorous physical activity (min/day)	37.9	23	42	23	35.7	23.8	35	21	
Energy prop. 5−11 am (%)	25.5	5.8	30.3	4.3	23	4	22.4	5.5	
Energy prop. 5 pm–12 am (%)	35.8	4.3	33.4	3.4	36.8	3.7	37.5	4.8	
Eating window (h)	11.5	0.6	11.6	0.5	11.8	0.4	11	0.5	
Pre-sleep fasting (h)	2.5	0.4	2.2	0.3	2.5	0.3	2.9	0.4	
Eating frequency (number of meals per day)	4.16	0.4	4.41	0.3	4.19	0.3	3.77	0.3	
Sex (*N*, %)									
Male	1,091	50	371	47	444	54	276	47	
Female	1,104	50	420	53	375	46	309	53	
Country (*N*, %)									
Italy	587	27	98	12	312	38	177	30	
Estonia	549	25	172	22	178	22	199	34	
Cyprus	171	8	64	8	68	8	39	7	
Belgium	176	8	116	15	38	5	22	4	
Sweden	233	11	116	15	75	9	42	7	
Germany	289	13	133	17	78	10	78	13	
Hungary	87	4	44	6	20	2	23	4	
Spain	103	5	48	6	50	6	5	1	
Weight status (*N*, %)									
Under weight	151	7	87	11	43	5	21		4
Normal weight	1,451	66	570	72	563	69	318		54
Overweight	421	19	108	14	144	18	169		29
Obese	172	8	26	3	69	8	77		13
ISCED (*N*, %)									
Low	1,081	49	280	35	471	58	330		56
High	1,114	51	511	65	348	42	255		44
Misreporters according to Goldberg cutoffs (*N*, %)									
No	1,755	80	717	91	665	81	373		64
Yes	440	20	74	9	154	19	212		36
Diabetes mellitus Type 2 (*N*, %)									
No	2,180	99	790	100	812	99	578		99
Yes	15	1	1	0	7	1	7		1
Puberty (*N*, %)									
No	1,221	58	635	84	376	47	210		37
Yes	898	42	119	16	424	53	355		63

**Table 2:** Mean and standard deviation (SD) of HOMA-IR, HbA1c, and triglycerides *z*-score by sex, country, ISCED, and weight status.

	HOMA-IR *z*-score		HbA1c *z*-score		Triglycerides *z*-score	
	Mean	SD	Mean	SD	Mean	SD
Sex						
Boys	0.2	1.2	0	0.9	0	1
Girls	0.2	1.2	0	1	0	1
Country						
Italy	0.5	1.2	0	1	0.1	1
Estonia	0.1	1.2	−0.1	1	0	1
Cyprus	0.1	1.2	0.2	0.9	0	1.1
Belgium	0.2	1.2	0.3	1	−0.2	1
Sweden	−0.4	1.3	−0.5	1.1	−0.1	1
Germany	0.3	1	0.1	0.8	0	0.9
Hungary	0.4	1.1	0.1	0.9	0.3	0.9
Spain	−0.2	1	−0.2	0.9	−0.3	1
ISCED						
Low	0.4	1.1	0	0.9	0.1	1.1
High	0	0.9	−0.1	0.9	−0.1	0.9
Weight status						
Under weight	−0.2	0.9	−0.1	1	−0.3	0.9
Normal weight	0	1.1	*−0.1*	1	−0.1	1
Overweight	0.6	1.2	0	1	0.3	1
Obese	1.4	1.2	0.3	1	0.7	1.1

**Table 3:** Mean *z*-scores of usual intake in the three meal timing pattern.

	“Early-often”	“Late-long”	“Late-infrequent-short”
Energy proportion 5−11 am	**0.83**	−0.42	*−0.53*
Energy proportion 5 pm–12 am	*−0.54*	0.23	**0.41**
Eating window	0.17	**0.53**	*−0.98*
Pre-sleep fasting	*−0.66*	0	**0.89**
Eating frequency	**0.64**	0.08	*−0.98*

The highest (lowest) value per row is marked bold (italic).

**Table 4:** Associations of the health outcomes HOMA-IR, HbA1c, and triglycerides with the meal timing derived from linear mixed models.

Model	Basic^a^			Puberty^b^			Energy and HFI^c^			Fully adjusted^d^		
Outcome (“early-often” as ref.)	*β*	95% CI		*β*	95% CI		*β*	95% CI		*β*	95% CI	
HOMA-IR *z*-score												
Late-long	0.06	−0.09	0.21	0.06	−0.10	0.21	0.06	−0.09	0.21	0.05	−0.1	0.21
Late-infrequent-short	0.19	0.01	0.36	0.19	−0.01	0.37	0.18	0	0.35	0.18	0	0.37
HbA1c *z*-score												
Late-long	−0.02	−0.15	0.11	−0.01	−015	0.12	−0.02	−0.15	0.11	−0.02	−0.15	0.12
Late-infrequent-short	−0.02	−0.17	0.14	−0.01	−0.18	0.15	−0.01	−0.17	0.14	−0.01	−0.17	0.15
Triglycerides *z*-score												
Late-long	0.02	−0.11	0.15	0.02	−0.11	0.15	0.02	−0.12	0.15	0.02	−0.13	0.16
Late-infrequent-short	0.05	−0.11	0.21	0.05	−0.11	0.21	0.03	−0.13	0.19	0.03	−0.13	0.2

^a^Adjusted for age, BMI *z*-score, ISCED, sex, country and family membership as random effect (*N* = 2,195 for HOMA-IR, *N* = 2,152 for HbA1c, *N* = 2,174 for triglycerides); ^b^additionally adjusted for puberty status (*N* = 2,119 for HOMA-IR, *N* = 2,077 for HbA1c, *N* = 2,099 for triglycerides); ^c^additionally adjusted for energy intake and healthy food intake (HFI) (*N* = 2,195 for HOMA-IR, *N* = 2,152 for HbA1c, *N* = 2,174 for triglycerides); ^d^additionally adjusted for energy intake, healthy food intake and sleep (*N* = 2,031 for HOMA-IR, *N* = 1,990 for HbA1c, *N* = 2,013 for triglycerides).

**Table 5:** Association between HOMA-IR *z*-score and selected exposures based on best subset selection analysis.

Model	Selected exposures	*β*	95% Confidence interval	
Basic^a^	Eating window (hr)	−0.16	−0.35	−0.11
(*N* = 2,195)	Energy proportion 5−11 am (%)	−0.01	−0.02	0
	Pre-sleep fasting (hr)	−0.15	−0.37	0
Energy + HFI^b^	Eating window (hr)	−0.19	−0.36	−0.13
(*N* = 2,195)	Energy proportion 5−11 am (%)	0.01	0	0.03
	Pre-sleep fasting (hr)	−0.14	−0.36	0.01
Fully adjusted^c^	Eating window (hr)	−0.21	−0.38	−0.14
(*N* = 2,031)	Energy proportion 5−11 am (%)	0.01	0	0.03
	Pre-sleep fasting (hr)	−0.15	−0.39	0.02

^a^Adjusted for age, BMI *z*-score, ISCED, sex, country and family membership as random effect; ^b^additionally adjusted for energy intake and healthy food intake (HFI); ^c^additionally adjusted for energy intake, healthy food intake, and sleep duration.

## Data Availability

The data that support the findings of this study are available from I.Family study centres but restrictions apply to the availability of these data and so are not publicly available. Data are however available from the authors upon reasonable request and with permission of the steering committee of the I.Family study.

## Abbreviations

24HDR: 24-hr dietary recall
BMI: Body mass index
CI: Confidence interval
CR: Circadian rhythm
EI: Energy intake
EPIC: European Prospective Investigation into Cancer and Nutrition
HbA1c: Glycated haemoglobin
HOMA-IR: Homoeostasis model assessment for IR
IR: Insulin resistance
ISCED: International Standard Classification of Education
MTP: Meal timing pattern
*N*: Number
NCI: National Cancer Institute
WE: Weekend
WD: Weekdays
yrs: Years
SACANA: Self-Administered Children, Adolescents, and Adult Nutrition Assessment
SD: Standard deviations.

## Supplementary Materials

Supplementary Materials Figure S1: Meal timing patterns of plausibly reporting children and adolescents (*N* = 1,755): “Early-often”, “Late-long,” and “Late-infrequent-short”. Cluster means of the three meal timing patterns are shown. The scale ranges from −1 to 1 with additional tick lines for −0.5, 0, and 0.5. Table S1: overview of meal times for breakfast, lunch and dinner in the eight different counties. Table S2: associations of the health outcomes HOMA-IR, HbA1c, and triglycerides with the meal timing patterns based on different linear mixed models in the plausibly reporting sample and sensitivity analysis: associations of the health outcomes HOMA, HbA1c, and triglycerides with the meal timing patterns based on different linear mixed models. Table S3: association between HbA1c *z*-score and selected exposures based on best subset selection. Table S4: association between triglycerides *z*-score and selected exposures based on best subset selection. Table S5: association between HOMA-IR *z*-score and selected exposures based on best subset selection analysis for plausible reporters only. Table S6: association between HbA1c *z*-score and selected exposures based on best subset selection analysis for plausible reporters only. Table S7: association between triglycerides *z*-score and selected exposures based on best subset selection analysis for plausible reporters only. Statistical details on the cluster analysis.
