# Supplementary material for: A Late Meal Timing Pattern Is Associated with Insulin Resistance in European Children and Adolescents
Source: Pediatr Diabetes. 2024 Mar 1;2024:6623357. doi: 10.1155/2024/6623357 (PMC12020773; doi:10.1155/2024/6623357)
Supplement: Supplementary Materials — Figure S1: Meal timing patterns of plausibly reporting children and adolescents (N = 1,755): “Early-often”, “Late-long,” and “Late-infrequent-short”. Cluster means of the three meal timing patterns are shown. The scale ranges from −1 to 1 with additional tick lines for −0.5, 0, and 0.5. Table S1: overview of meal times for breakfast, lunch and dinner in the eight different counties. Table S2: associations of the health outcomes HOMA-IR, HbA1c, and triglycerides with the meal timing patterns based on different linear mixed models in the plausibly reporting sample and sensitivity analysis: associations of the health outcomes HOMA, HbA1c, and triglycerides with the meal timing patterns based on different linear mixed models. Table S3: association between HbA1c z-score and selected exposures based on best subset selection. Table S4: association between triglycerides z-score and selected exposures based on best subset selection. Table S5: association between HOMA-IR z-score and selected exposures based on best subset selection analysis for plausible reporters only. Table S6: association between HbA1c z-score and selected exposures based on best subset selection analysis for plausible reporters only. Table S7: association between triglycerides z-score and selected exposures based on best subset selection analysis for plausible reporters only. Statistical details on the cluster analysis. [file 6623357.f1.docx]

**Figure SM1**. Meal timing patterns of children and adolescents with plausible dietary reports only (N=1755): “Early-often type”, “Late-long type” and “Late-infrequent-short type” cluster means are shown. The scale ranges from -1 to 1 with additional tick lines for every 0.5 increment (-0.5, 0 and 0.5).

**Table SM1.** Overview of meal times for breakfast, lunch and dinner in the eight different counties

| **Country** | **Meal** | **Meal times** |  |  |  |
| --- | --- | --- | --- | --- | --- |
|  |  | **10th percentile** | **Mean** | **Median** | **90th percentile** |
| **Italy** | Breakfast | 07:00 | 08:02 | 07:45 | 09:45 |
|  | Lunch | 13:00 | 13:32 | 13:30 | 14:00 |
|  | Dinner | 19:30 | 20:20 | 20:30 | 21:30 |
| **Estonia** | Breakfast | 06:45 | 08:05 | 07:30 | 10:15 |
|  | Lunch | 10:30 | 12:44 | 12:30 | 15:00 |
|  | Dinner | 17:15 | 18:56 | 19:00 | 20:45 |
| **Cyprus** | Breakfast | 06:45 | 08:10 | 08:00 | 10:00 |
|  | Lunch | 13:00 | 13:41 | 13:30 | 14:45 |
|  | Dinner | 19:00 | 19:46 | 20:00 | 21:00 |
| **Belgium** | Breakfast | 07:00 | 08:07 | 08:00 | 09:30 |
|  | Lunch | 12:00 | 12:26 | 12:15 | 13:00 |
|  | Dinner | 17:30 | 18:39 | 18:30 | 20:00 |
| **Sweden** | Breakfast | 06:45 | 07:55 | 07:30 | 10:00 |
|  | Lunch | 11:00 | 12:13 | 12:00 | 14:00 |
|  | Dinner | 16:30 | 18:07 | 18:00 | 19:45 |
| **Germany** | Breakfast | 06:30 | 08:18 | 08:00 | 10:30 |
|  | Lunch | 12:30 | 13:39 | 13:30 | 15:00 |
|  | Dinner | 18:00 | 19:01 | 19:00 | 20:00 |
| **Hungary** | Breakfast | 06:30 | 07:48 | 07:30 | 09:30 |
|  | Lunch | 12:00 | 12:52 | 12:45 | 14:00 |
|  | Dinner | 18:00 | 18:51 | 19:00 | 20:00 |
| **Spain** | Breakfast | 07:00 | 08:27 | 08:15 | 10:15 |
|  | Lunch | 13:00 | 14:02 | 14:15 | 15:15 |
|  | Dinner | 20:30 | 21:05 | 21:15 | 22:15 |

**Table SM2A**. Associations of the health outcomes HOMA, HbA1c and triglycerides with the meal timing patterns in the plausible reporters derived fromthree linear mixed models

| **Model** | |  | **Basic^a^** | | | **Energy &HFI^b^** | | | **Fully adjusted^c^** | | |
| --- | --- | --- | --- | --- | --- | --- | --- | --- | --- | --- | --- |
| **Outcome (“Early-often” as ref.)** | | | **β** | **95% CI** | | **β** | **95% CI** | | **β** | **95% CI** | |
| **HOMA z-score** | Late Long | | 0.02 | -0.14 | 0.19 | 0.03 | -0.13 | 0.18 | 0.03 | -0.13 | 0.2 |
|  | Late-infrequent-short | | 0.13 | -0.05 | 0.3 | 0.12 | -0.04 | 0.29 | 0.11 | -0.06 | 0.29 |
| **HbA1c z-score** | Late Long | | -0.01 | -0.17 | 0.15 | -0.01 | -0.17 | 0.15 | 0 | -0.16 | 0.15 |
|  | Late-infrequent-short | | -0.01 | -0.17 | 0.16 | -0.01 | -0.18 | 0.16 | 0 | -0.16 | 0.16 |
| **Triglycerides z-score** | Late Long | | 0.01 | -0.14 | 0.17 | 0.01 | -0.14 | 0.16 | 0.02 | -0.14 | 0.18 |
|  | Late-infrequent-short | | 0.02 | -0.14 | 0.18 | 0.02 | -0.14 | 0.18 | 0.03 | -0.14 | 0.19 |

^a^adjusted for age, BMI z-score, ISCED, sex, country and family membership as random effect (N=1755 for HOMA-IR, N = 1724 for HbA1c, N = 1738 for triglycerides);

^b^ additionally adjusted for energy intake and healthy food intake (HFI)(N=1755 for HOMA-IR, N = 1724 for HbA1c, N = 1738 for triglycerides);

^c^ additionally adjusted for energy intake, HFI and sleep (N=1628 for HOMA-IR, N = 1599 for HbA1c, N = 1614 for triglycerides)

**Tab SM2B**. Sensitivity analysis: associations of the health outcomes HOMA, HbA1c and triglycerides with the meal timing patterns based on different linear mixed models considering moderate-to-vigorous physical activity and an overweight/obesity interaction term

| **Model** |  | **MVPA model^a^** | |  | **Overweight/obesity interaction model^b^** | | | | |  |
| --- | --- | --- | --- | --- | --- | --- | --- | --- | --- | --- |
|  |  |  |  |  | **Pattern** |  |  | **Pattern:overweight/obesity** | | |
| **Outcome (“Early-often” as ref.)** | | **β** | **95-%-CI** |  | **β** | **95-%-CI** |  | **β** | **95-%-CI** |  |
| **HOMA z-score** | **Late Long** | 0,07 | -0,12 | 0,25 | 0,09 | -0,08 | 0,26 | -0,02 | -0,39 | 0,34 |
|  | **Late-infrequent-short** | 0,18 | -0,04 | 0,40 | 0,20 | -0,01 | 0,40 | 0,05 | -0,35 | 0,46 |
| **HbA1c z-score** | **Late Long** | -0,03 | -0,19 | 0,13 | -0,02 | -0,16 | 0,13 | 0,02 | -0,29 | 0,32 |
|  | **Late-infrequent-short** | -0,02 | -0,22 | 0,17 | -0,01 | -0,19 | 0,17 | 0 | -0,35 | 0,34 |
| **Triglycerides z-score** | **Late Long** | 0,03 | -0,14 | 0,19 | 0,03 | -0,12 | 0,18 | -0,02 | -0,33 | 0,30 |
|  | **Late-infrequent-short** | 0,05 | -0,15 | 0,26 | 0,07 | -0,12 | 0,26 | -0,03 | -0,38 | 0,33 |

**^a^** MVPA model: adjusted for age, BMI z-score, ISCED, sex, country, MVPA and family membership as random effect (N=1457 for HOMA-IR, N = 1429 for HbA1c, N = 1448 for triglycerides)

**^b^** overweight interaction model: additionally adjusted for overweight instead of BMI z-score and considering interaction effect pattern:overweight/obesity (N=2195 for HOMA-IR, N = 2152 for HbA1c, N = 2174 for triglycerides)

**Table SM3**. Association between HbA1c z-score and selected exposures based on best subset selection analysis.

| **Model** |  | **β** | **95% CI** | |
| --- | --- | --- | --- | --- |
| **Basic^a^** | Energy proportion 5-11 am (%) | 0.01 | 0 | 0.02 |
| **Energy + HFI** | Energy proportion 5-11 am (%) | 0.01 | 0 | 0.02 |
| **Fully adjusted^c^** | Energy proportion 5-11 am (%) | 0.01 | 0 | 0.02 |

^a^adjusted for age, BMI z-score, ISCED, sex, country and family membership as random effect;

^b^ additionally adjusted for energy intake and healthy food intake (HFI);

^c^ additionally adjusted for energy intake, HFI and sleep duration

**Table SM4**. Association between triglycerides z-score and selected exposures based on best subset selection.

| **Model** |  | **β** | **95%CI** | |
| --- | --- | --- | --- | --- |
| **Basic^a^** | Energy proportion 5 pm - 12 am (%) | 0.01 | 0 | 0.02 |
| **Energy + HFI^b^** | Energy proportion 5-11 am (%) | 0 | -0.02 | 0 |
| **Fully adjusted^c^** | Energy proportion 5 pm - 12 am (%) | 0 | -0.01 | 0.02 |

^a^ adjusted for age, BMI z-score, ISCED, sex, country and family membership as random effect;

^b^ additionally adjusted for energy intake and healthy food intake (HFI);

^c^ additionally adjusted for energy intake, healthy food intake and sleep duration

**Table SM5**. Association between HOMA z-score and selected exposures based on best subset selection analysis for plausible reporters only.

| **Model** | **Selected exposures** | **β** | **95% CI** | |
| --- | --- | --- | --- | --- |
| **Basic^a^** | Eating window (h) | -0.19 | -0.45 | -0.1 |
|  | Energy proportion 5-11 (%) | -0.01 | -0.02 | 0 |
|  | Pre-sleep fasting (h) | -0.25 | -0.65 | 0.06 |
| **Energy + HFI^b^** | Eating window (h) | -0.19 | -0.45 | -0.09 |
|  | Energy proportion 5-11 (%) | -0.01 | -0.02 | 0 |
|  | Pre-sleep fasting (h) | -0.2 | -0.57 | 0.15 |
| **Fully adjusted^c^** | Eating window (h) | -0.24 | -0.5 | -0.12 |
|  | Pre-sleep fasting (h) | -0.24 | -0.58 | 0.14 |

^a^adjusted for age, BMI z-score, ISCED, sex, country and family membership as random effect;

^b^ additionally adjusted for energy intake and healthy food intake (HFI);

^c^ additionally adjusted for energy intake, healthy food intake and sleep duration

**Table SM6**. Association between HbA1c z-score and selected exposures based on best subset selection analysis for plausible reporters only.

| **Model** |  | **β** | **95% CI** | |
| --- | --- | --- | --- | --- |
| **Basic^a^** | Eating window (h) | 0.07 | -0.08 | 0.16 |
| **Energy + HFI^b^** | Eating window (h) | 0.06 | -0.08 | 0.15 |
| **Fully adjusted^c^** | Presleep fasting (h) | -0.12 | -0.44 | 0.11 |

^a^adjusted for age, BMI z-score, ISCED, sex, country and family membership as random effect;

^b^ additionally adjusted for energy intake and healthy food intake;

^c^ additionally adjusted for energy intake, healthy food intake (HFI) and sleep duration

**Table SM7**. Association between triglycerides z-score and selected exposures based on best subset selection analysis for plausible reporters only.

| **Model** |  | **β** | **95% CI** | |
| --- | --- | --- | --- | --- |
| **Basic^a^** | Energy proportion 5-11 (%) | -0.01 | -0.02 | 0 |
| **Energy + HFI^b^** | Energy proportion 5-11 (%) | 0 | -0.02 | 0 |
| **Fully adjusted^c^** | Energy proportion 5-11 (%) | 0 | -0.02 | 0 |

^a^adjusted for age, BMI z-score, ISCED, sex, country and family membership as random effect;

^b^ additionally adjusted for energy intake and healthy food intake (HFI);

^c^ additionally adjusted for energy intake, healthy food intake and sleep duration

**Statistical details on the cluster analysis**

In the appendix we give details on the clustering procedure to derive the final meal timing patterns. We considered the k-means algorithm by Hartigan and Wong (1) and the Gaussian mixture model (GMM) (2) as cluster methods and the z-scores of the usual energy proportion (5-11 am), the usual energy proportion (5 pm - 12am), usual eating window, usual pre-sleep fasting and the usual eating frequency as cluster variables. For both cluster method solutions with C = 3, 4 or 5 clusters were compared. Since the k-means algorithm only converges to a local minimum the algorithm for a fix C was applied with 10 random starts and the corresponding solution with the lowest total within cluster sum of square was selected. For the GMM, models with identical covariance matrices and equal variances were chosen to provide a simple geometric interpretation of cluster solutions.

The six cluster solutions were compared regarding total within cluster sum of squares, silhouette coefficients (3) and reproducibility of cluster solutions (4). The total within sums of squares and silhouette coefficients did not give a clear evidence for a superior cluster solution. The reproducibility was investigated using 100 random samples of 80% of the total sample for cluster analysis. The solutions were compared with the corresponding solutions of the whole sample by adjusted Rand Index (aRI) (5) and misclassification rate (MR). The resulting mean aRIs were 0.90, 0.87 and 0.85, the mean MRs were 0.04, 0.05 and 0.06 for the k-mean algorithm with C = 3, 4 and 5 clusters, respectively. The resulting mean aRIs were 0.75, 0.78 and 0.61, the mean MRs were 0.09, 0.08 and 0.18, for the GMM with C = 3, 4 and 5 clusters, respectively. That means regarding reproducibility the k-means cluster solution with C = 3 clusters was superior. Since the interpretability of this solution was also given we selected it as final cluster solution for further investigations.
